# Pharmacokinetics of caffeine administered in a time-release versus regular tablet form

**DOI:** 10.1186/1550-2783-11-S1-P23

**Published:** 2014-12-01

**Authors:** Adam M Gonzalez, Jay R Hoffman, Adam J Wells, Gerald T Mangine, Jeremy R Townsend, Adam R Jajtner, Ran Wang, Amelia A Miramonti, Gabriel J Pruna, Michael B LaMonica, Jonathan D Bohner, Mattan W Hoffman, Leonardo P Oliveira, David H Fukuda, Maren S Fragala, Jeffrey R Stout

**Affiliations:** 1 University of Central Florida, Orlando, Florida, USA

## Background

Caffeine has been associated with enhancing the ability to perform mental tasks and elevate feelings of energy, however, a single dose of caffeine typically induces only 90-120 minutes of increased alertness and is often associated with an acute “crash” state following its metabolism. The nature of formulation can directly influence the rate and extent of absorption following oral administration. Time-release caffeine supplements have been developed to prolong the effects of caffeine. The purpose of this study was to compare the plasma caffeine pharmacokinetics following ingestion of a time-release caffeine capsule (TR-CAF) to an equivalent dose of a regular caffeine capsule (CAF) and a placebo (PL) over an 8-hour period.

## Methods

Ten healthy males (25.9 ± 3.2 y; 181.3 ± 8.2 cm; 92.9 ± 9.9 kg; 13.3 ± 3.6 % body fat) who regularly consume caffeine volunteered to participate in this double-blind, placebo-controlled, cross-over study. Participants were randomized into three experimental trials: Ingestion of EnergizeTM; CAF; and PL. The EnergizeTM supplement contained 194 mg time-release caffeine and CAF contained the equivalent amount of regular caffeine. PL contained rice flour only. Blood draws occurred at baseline and at every hour during the 8-hour period. Participants were provided a standardized breakfast and lunch and were permitted to drink water ad libitum. Plasma caffeine concentrations were quantified using high performance liquid chromatography (HPLC). Consent to publish the results was obtained from all participants.

## Results

Plasma caffeine concentrations for both caffeine supplements were significantly greater than PL over the 8-hour study duration. CAF rapidly reached peak plasma caffeine concentration (2.40 ± 0.40 mg•L-1) at 3 hours following ingestion, while TR-CAF reached peak plasma caffeine concentration (1.88 ± 0.46 mg•L-1) at 6 hours following ingestion. Plasma caffeine concentrations were significantly greater (p<0.05) in CAF compared to TR-CAF during hours 2-5. However, plasma caffeine concentrations were significantly greater (p=0.042) in TR-CAF compared to CAF at hour 8. AUC analysis showed plasma caffeine concentrations for CAF were significantly greater than TR-CAF for hours 1-4 (p<0.0001) and over the 8-hour study duration (p<0.001).

## Conclusion

The results suggest that the rate of caffeine absorption following TR-CAF was slower and more sustained as compared to CAF over 8 hours following ingestion which may prolong the effects of caffeine and limit the acute “crash” state.

